# Biological Brain Age Prediction Using Cortical Thickness Data: A Large Scale Cohort Study

**DOI:** 10.3389/fnagi.2018.00252

**Published:** 2018-08-22

**Authors:** Habtamu M. Aycheh, Joon-Kyung Seong, Jeong-Hyeon Shin, Duk L. Na, Byungkon Kang, Sang W. Seo, Kyung-Ah Sohn

**Affiliations:** ^1^Department of Software and Computer Engineering, Ajou University, Suwon, South Korea; ^2^School of Biomedical Engineering, Korea University, Seoul, South Korea; ^3^Department of Neurology, Samsung Medical Center, School of Medicine, Sungkyunkwan University, Seoul, South Korea; ^4^Neuroscience Center, Samsung Medical Center, Seoul, South Korea; ^5^Department of Health Sciences and Technology, Clinical Research Design and Evaluation, SAIHST, Sungkyunkwan University, Seoul, South Korea

**Keywords:** aging, cortical thickness, cortical lobe, regression analysis, ROI, Sparse Group Lasso, Gaussian process

## Abstract

Brain age estimation from anatomical features has been attracting more attention in recent years. This interest in brain age estimation is motivated by the importance of biological age prediction in health informatics, with an application to early prediction of neurocognitive disorders. It is well-known that normal brain aging follows a specific pattern, which enables researchers and practitioners to predict the age of a human's brain from its degeneration. In this paper, we model brain age predicted by cortical thickness data gathered from large cohort brain images. We collected 2,911 cognitively normal subjects (age 45–91 years) at a single medical center and acquired their brain magnetic resonance (MR) images. All images were acquired using the same scanner with the same protocol. We propose to first apply Sparse Group Lasso (SGL) for feature selection by utilizing the brain's anatomical grouping. Once the features are selected, a non-parametric non-linear regression using the Gaussian Process Regression (GPR) algorithm is applied to fit the final age prediction model. Experimental results demonstrate that the proposed method achieves the mean absolute error of 4.05 years, which is comparable with or superior to several recent methods. Our method can also be a critical tool for clinicians to differentiate patients with neurodegenerative brain disease by extracting a cortical thinning pattern associated with normal aging.

## Introduction

Aging is a biological process that exhibits distinct attributes from childhood to old age. Human brain aging is affected by progressive and regressive neuronal processes due to cell growth and death (Silk and Wood, 2011). Moreover, environmental factors and health conditions affect structural changes in the brain (Pannacciulli et al., 2006; Chee et al., 2009; Ziegler et al., 2012). Thus, the structure of the brain changes continuously throughout a life span. Human brain degeneration has a specific pattern during the normal aging process (Seidman et al., 2004; Fjell et al., 2009; Fjell and Walhovd, 2010). This provided the groundwork for studies that predict brain age from brain atrophy patterns. The majority of these studies were inspired by the clinical benefits of biological brain age estimation for early prediction of neurocognitive disorders. For example, diseases related to Alzheimer's that change brain aging patterns can be examined. The human brain changes over a lifespan. According to neuroscience studies, the brain can be macro-anatomically grouped into the following major six lobes: frontal, temporal, parietal, limbic, occipital, and insula (Allen et al., 2005). Cortical thickness rate of decline in an elderly lifespan is variable in each cortex lobe (Resnick et al., 2003; Seidman et al., 2004; Allen et al., 2005; Fjell et al., 2009; Fjell and Walhovd, 2010; Lemaitre et al., 2012; Ziegler et al., 2012; Ruigrok et al., 2014). These studies identified that there is variability in the brain aging process from person to person, and among age groups (neonatal, youth and adult ages). These evidences indicate the variability of anatomical measurement trajectories in different cortical regions. The demographic variables of education and gender are also confounding factors that influence cortical thickness (Tang et al., 2013; Li et al., 2014; Mortby et al., 2014; Ruigrok et al., 2014; Ritchie et al., 2017; Belathur Suresh et al., 2018; Thow et al., 2018). In particular, the recent work in Belathur Suresh et al. (2018) and Thow et al. (2018) demonstrated the impact of education on cortical thickness for the accuracy of Alzheimer's disease detection. These indicate the potentiality of gender and education as predictive variables in addition to brain anatomical features. We also include these demographic variables in our study.

Brain age prediction is an active research area. There have been continuous research efforts in the estimation of human biological brain age using magnetic resonance imaging (MRI). Currently, there are two major trends of using brain MRI to predict age: (1) using raw image data and (2) cortex anatomical measures. The acquisition of both data sources are related to the arguments for and against using surface-based or voxel-based registration methods as described in Clarkson et al. (2011). The works in both directions are continually improving and have significantly assisted practitioners and researchers in the neurology-related research domain. Progressively, interesting and fruitful brain-age-prediction analysis results are presented in Ashburner (2007), Franke et al. (2010), Cole et al. (2015, 2017), Kondo et al. (2015), Wang et al. (2015), Alam et al. (2016), Cherubini et al. (2016), Cole and Franke (2017), Liem et al. (2017), Valizadeh et al. (2017), and Madan and Kensinger (2018). Recently, the prediction of brain age using 3D raw image data using deep learning presented by Cole et al. (2017) showed a promising result. Cole et al. (2017) used convolutional neural network (CNN) algorithms and obtained the best mean absolute error (MAE) of 4.16 years. This result is comparably an improvement on their prior work of brain age prediction using Gaussian Process Regression (GPR), which had an MAE = 4.66 years (Cole et al., 2015). The prediction of brain age prediction using surface-based features has also been studied (Kondo et al., 2015; Wang et al., 2015; Liem et al., 2017; Valizadeh et al., 2017; Madan and Kensinger, 2018). The recent study by Madan and Kensinger (2018) compared different parcellation approaches to extract explanatory features for brain age prediction from MRIs. They reported the median absolute error (MdAE) = 6–7 years using Relevant Vector Regression (RVR). This prediction result is obtained by the combination of cortical thickness and fractal dimension. Another recent study by Valizadeh et al. (2017) presented a detailed feasibility analysis of age prediction from surface-based measures. They described brain age prediction using anatomical measures such as cortical thickness, surface area, cortical volume, and their combinations from a human brain MRI using 148 regional cortex compartments. Their overall analysis showed the plausibility of age prediction from brain surface-based features with high accuracy. In it, the best performance was obtained using a neural network prediction model, where the prediction errors were similar to prior results reported in Wang et al. (2015) and Cherubini et al. (2016). The analysis by Valizadeh et al. (2017) revealed an additional important point that prediction error increases with increasing age specifically in older adults. Wang et al. (2015) used an RVR model (Tipping, 2001) to estimate age on the basis of different anatomical measures such as cortical surface area, cortical thickness, mean curvature, Gaussian curvature, and a combination of these measures by using 148 regions of interest (ROIs). The best performance result obtained was by a combination of cortical thickness and the curvature predictive features, with a reported root mean square error (RMSE) of 5.57 years. The findings of Wang et al. (2015) support the idea that among surface-based features, cortical thickness is more informative for age-related morphometric changes across the life span than other type of features. The age prediction algorithm reported in Kondo et al. (2015) also used RVR based on a local feature extraction approach from a T1-weighted MRI. They used 90 local regions of white matter, gray matter, and cerebrospinal fluid (CSF) to reduce the requirement for high-order features when combining brain anatomical features for age estimation to simplify the medical implications. There are also several studies that have investigated the potential of functional connectivity measures derived from resting state functional MRI (rsfMRI) data for the brain age prediction (Dosenbach et al., 2010; Vergun et al., 2013; Smyser et al., 2016; Liem et al., 2017). In these studies, functional connectivity measures were derived from rsfMRI data based on regions of interest (ROIs) defined by different parcellation methods and the support vector regression was adopted to build age prediction models.

In general, the major components of brain age estimation from MRI are feature extraction, feature selection or identification of the explanatory variables from a brain feature dataset, and the regression model for fitting the target age. According to state-of-the-art studies, a consistent age-to-brain-development relationship pattern is exhibited using surface-based brain features (Clarkson et al., 2011; Madan and Kensinger, 2018). Further, model overfitting is one of the challenging issues in brain age estimation using cortical measures due to the inherent correlation among brain features. Wang et al. (2015) and Valizadeh et al. (2017) employed principal component analysis (PCA) for dimension reduction and feature extraction. Kondo et al. (2015) used a local feature extraction approach. For model fitting, the majority of the works focused on kernel-based regression, specifically RVR models for the prediction of age from brain anatomical features. The recent implementation of deep learning algorithms also benefits from the automatic feature learning property of the algorithm when the sample size is sufficient (Cole et al., 2017).

In this paper, we propose the prediction of brain age based on cortical thickness data by first applying Sparse Group Lasso (SGL) (Simon et al., 2013) for selecting important features from each major cortical lobe and then using GPR (Rasmussen and Williams, 2006) for fitting the age prediction model. The rate of decline in cortical thickness can differ in each cortex lobe. Human aging is related to a healthy brain-change pattern within the respective cortical lobes. SGL is a regularized regression method for grouped variables that supports feature selection on a group level and within group level. As such, SGL is robust and consistent in feature selection. Thus, SGL is an appropriate approach to select explanatory features on and within the cortical lobes. Then, we deploy GPR, which is a non-parametric non-linear regression method to predict the target subject's brain age based on the selected features. We obtained prediction accuracy of MAE = 4.05 years by using the proposed method, which is comparable with or superior to several recent methods.

## Methods

### Study participants

Study participants were recruited from the Health Promotion Center of Samsung Medical Center (SMC), Seoul, Korea. The study population was comprised of men and women 45 years of age or older who underwent a comprehensive health screening exam between January 1, 2009 and December 31, 2014. Of the eligible participants 3,370 attended a preventative medical check-up, which included an assessment of cognitive function and dementia status. All study participants underwent a high-resolution 3.0 Tesla (3T) brain MRI, which included three-dimensional (3D) volume images as a part of their dementia assessment. The assessment procedure used for the participants has been described in detail elsewhere (Lee et al., 2016b). Participants were excluded for meeting disqualifying conditions: 202 participants with missing educational data or missing Mini-Mental State Examination score (MMSE); 178 participants who showed significant cognitive impairment defined by MMSE scores below the 16th percentile in age-, sex-, and education-matched norms, or through an interview conducted by a qualified neurologist; and 136 participants with unreliable analyses of cortical thickness due to head motion, blurring of the MRI, inadequate registration to a standardized stereotaxic space, misclassification of tissue type, or inexact surface extraction, for which the image preprocessing and cortical thickness computation process were manually checked and corrected by an expert neuroanatomist. Participants were excluded if they had a cerebral, cerebellar, or brainstem infarction; hemorrhage; brain tumor; hydrocephalus; severe cerebral white matter hyperintensities (deep white matter ≥2.5 cm and caps or band ≥1.0 cm); or severe head trauma by personal history. The final sample size was 2,911 healthy individuals (1,460 males and 1,451 females). All 2,911 participants underwent a 3T brain MRI using the same type of scanner with the same scan parameters. We parcellated the cerebral cortex into 148 cortical ROIs based on the Destrieux Atlas (http://surfer.nmr.mgh.harvard.edu/fswiki/CorticalParcellation). For each of the 148 cortical ROIs, the average cortical thickness was computed. Because we learn the prediction model for brain age based on the chronological ages of healthy individuals, we further detect and exclude outlier samples to minimize the potential bias from individuals with unknown health conditions. After excluding noise and outliers (as is explained in section Filtering Outliers From Cortical Thickness Data), 2,705 observations (1,368 males and 1,337 females) remained. The age range of the subjects in this study was 45–91 years. The mean age of the subjects was 64.2 years, with a standard deviation of 7.1 years (male: 65.2 ± 6.9 years; female: 63.1 ± 7.2 years). See Table 1 for more details.

**Table 1:** Demographic characteristics of study participants.

**Age range (in years)**	**Number of participants**			**Education in years of study (mean ± SD)**		
	**Male**	**Female**	**Total**	**Male**	**Female**	**Total**
45–49	20	33	53	15.10 ± 2.57	13.76 ± 2.81	14.26 ± 2.77
50–54	63	118	181	14.17 ± 2.63	13.03 ± 3.59	13.43 ± 3.32
55–59	146	252	398	13.93 ± 13.93	12.24 ± 12.42	12.86 ± 12.83
60–64	423	410	833	14.16 ± 3.40	12.13 ± 3.40	13.16 ± 3.87
65–69	381	256	637	14.17 ± 3.95	10.79 ± 4.73	12.81 ± 4.59
70–74	210	171	381	14.00 ± 3.79	9.95 ± 4.74	12.18 ± 4.69
75–79	90	82	172	13.81 ± 4.43	8.71 ± 4.97	11.38 ± 5.34
80–84	31	13	44	15.68 ± 3.56	8.23 ± 6.06	13.48 ± 5.56
85–91	4	2	6	11.00 ± 7.57	9.00 ± 4.24	10.33 ± 6.25
**Total**	**1,368**	**1,337**	**2,705**	**14.13 ± 3.67**	**11.48 ± 4.44**	**12.82 ± 4.28**

This study was approved by the Institutional Review Board at the Samsung Medical Center. The requirement for informed consent was waived, as we only used de-identified data collected for clinical purposes during the health screening exams.

### Image acquisition and preprocessing

3D T1-weighted Turbo Field Echo MRI images were acquired from all participants in this study using the Philips 3T Achieva MRI scanner with the same imaging parameters (sagittal slice thickness 1.0 mm, over contiguous slice acquisition with 50% overlap; no gap; repetition time 9.9 ms; echo time 4.6 ms; flip angle 8°; and matrix size 240 × 240 reconstructed to 480 × 480 over a 240-mm field of view).

For each subject, we first performed image preprocessing using FreeSurfer v5.1.0 (Athinoula A. Martinos Center at the Massachusetts General Hospital, Harvard Medical School; http://surfer.nmr.mgh.harvard.edu/). FreeSurfer was used to volumetrically segment and parcellate cortex from T1-weighed images (Dale et al., 1999; Fischl et al., 1999, 2002; Desikan et al., 2006; Destrieux et al., 2010; Fischl, 2012; Klein and Tourville, 2012). We first constructed the outer and inner cortical surface meshes from the MR volume of each subject. The two meshes are isomorphic with the same vertices and connectivity because the outer surface was constructed by deforming the inner surface. In order to establish inter-subject correspondence, we resampled each subject's cortical surface to 40,962 vertices for each hemisphere using the previously proposed method (Cho et al., 2012).

For smoothing cortical thickness data, we adopted the noise removal procedure proposed by Cho et al. (2012) to our problem setting. Cho et al. (2012) employed the manifold harmonic transform (MHT) to delineate the cortical thickness data with its spatial frequency components (Vallet and Lévy, 2008). For the transform, the Laplace-Beltrami operator is used to obtain basis functions which results in robustness to noise by filtering out high frequency (Cho et al., 2012). Since high frequency components of the transformed cortical thickness data were regarded as noise, those components are filtered out, and the cortical thickness data were then reconstructed using only low frequency components (Chung et al., 2007).

In general, the mean cortical thickness values of 148 distinct ROIs were computed from each brain MRIs and used as predicting variables. The confounding variables of gender and education of the subjects were also included to these predictors because they have cortical thinning effect in relation to normal aging (Tang et al., 2013; Li et al., 2014; Mortby et al., 2014; Ruigrok et al., 2014; Ritchie et al., 2017; Belathur Suresh et al., 2018; Thow et al., 2018). The gender feature is a “0–1” binary variable that indicates whether the subject is male (0) or female (1). The education feature is a numeric value that reflects the level of education the subject has attained, which is related to the number of years of study (zero indicates uneducated, and a higher number of years of study indicates a higher level of education). Thus, we had 150 explanatory variables.

### Filtering outliers from cortical thickness data

In this study, we used mean cortical thickness data extracted from brain images for brain age prediction. The proposed model is a supervised learning method and it is understandable that the response variable is a chronological age, which is set under the assumption that chronological age and brain age is the same for a healthy subject. Outliers significantly affect the prediction accuracy of a model when non-robust statistical methods are used. Nevertheless, the cortical thickness measures can deviate from the expected range due to overlooked health factors, life style conditions, environmental effects and other related factors that affect cortical thickness. Most importantly, we are interested in investigating the outliers due to sample subjects that could be included in our target study due to bias or latent conditions of the cognitive health assessment. There are high risk factors related to cognitive health in older adults. Thus, our target sample (age 45–91 years) requires additional attention regarding the reliability of the subjects' cognitive health. Accordingly, we are interested in investigating the effects of outliers in the dataset by using an outlier-filtering method suitable to our problem data representation.

The choice of the outlier detection method predominantly depends on the nature and representation of the dataset, which requires an insight investigation of the problem domain such as small perturbation values. In this study, we adopted the Local Outlier Factor (LOF) method to our dataset (Breunig et al., 2000). LOF is a density-based unsupervised outlier detection method that has a property of comparing outliers to their local neighborhoods instead of the global data distribution. The age range in our study was from 45 to 91 years. In this age range, cortex thickness gradually decreases with increasing age. Thus, LOF can be used to identify outliers by grouping ages to their proximity. Accordingly, we grouped ages by eight intervals: 45–49, 50–54, … Then, LOF was applied on each interval to identify outliers. We are interested in checking outliers per age group interval because the cortical thickness changes gradually and we need to manage expected variabilities between the youngest age groups (approximately age 45) and the oldest age groups (around age 91) in the target sample of age range from 45 to 91 years.

In our dataset, we had *N* = 2,911 subjects and *p* = 148 features (mean cortical thickness values) for each subject. The demographic features, gender and education, were not included in this case. We check outliers in each subsets of the 2,911 observations per the specified age group. That is, we had sub-datasets, *S*_*n*_*i*_ × *p*_ where *n*_*i*_ is the number of observations in the given age group. Based on this, the outlier score values were computed using the LOF algorithm as stated in Breunig et al. (2000). LOF uses the K-nearest neighborhood approach to compute the outlier scores by setting k heuristically. The algorithm compares the density of each point to the density of its K-closest neighbors. Note that the distance between two points is the distance between two vectors $X_{1}\in {\mathbb{R}}^{p}$ and $X_{2}\in {\mathbb{R}}^{p}$. A higher value of LOF score indicates a potential outlier. An illustration of our proposed outlier filtering approach is displayed in Figure 2. After outlier filtering, 2,705 samples remained for the training set and testing set. The number of identified outliers for each age group is shown in Figure 3.

### Brain age modeling methods

Brain age prediction is a supervised regression problem. The response variable is chronological age. The predicting variables are mean cortical thickness measures of the 148 ROIs and the confounding factors of gender and education. The main challenge of brain age prediction from brain anatomical features is overfitting due to the correlation among brain features. Thus, the parsimony of the model is crucial for the analysis of the prediction and inferences. Balancing this tradeoff between bias and variance helps to overcome the overfitting problem in relation to analyzing the explanatory variables used in modeling the brain age prediction. Accordingly, the main concern of our approach is selecting the most important prediction variables from the existing features while maintaining good generalization.

**Figure 1:**
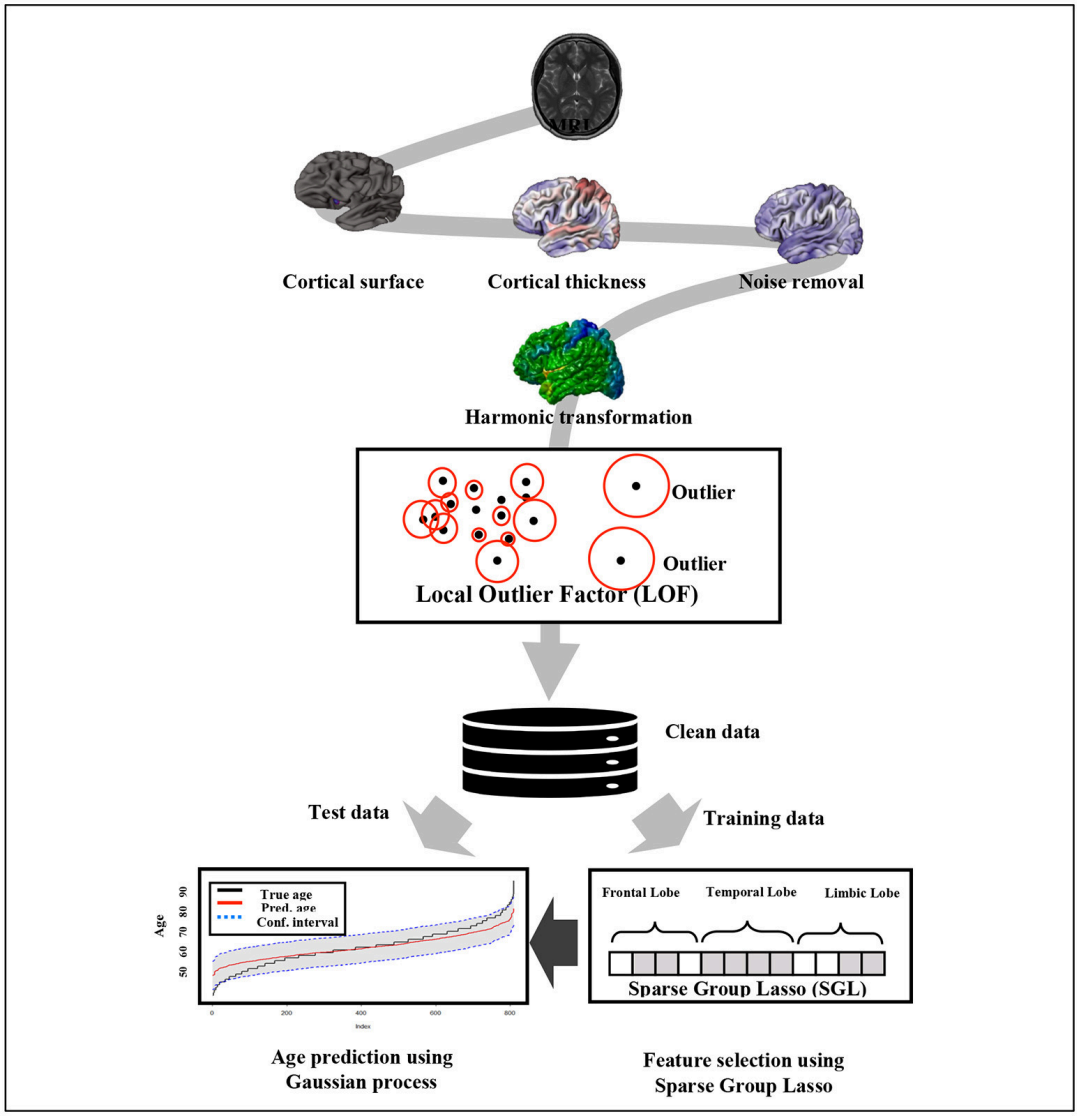
Overview of the proposed method.

The prediction accuracy of brain age using the given *P*-dimensional covariates can be improved by combining the individual strengths of the learning algorithms. The macro anatomical grouping of cortex structure into cerebral lobes favors the SGL model implementation. We combine the SGL that selects the most important features on a group and within group levels with the acceptable prediction power of kernel methods such as GPR. In general, we use the SGL model described in section Sparse Group Lasso to select the top *q* important features from the *P* covariates. Then, the brain age prediction model is fitted using GPR. The general framework of the proposed brain age prediction model is depicted in Figure 1.

**Figure 2:**
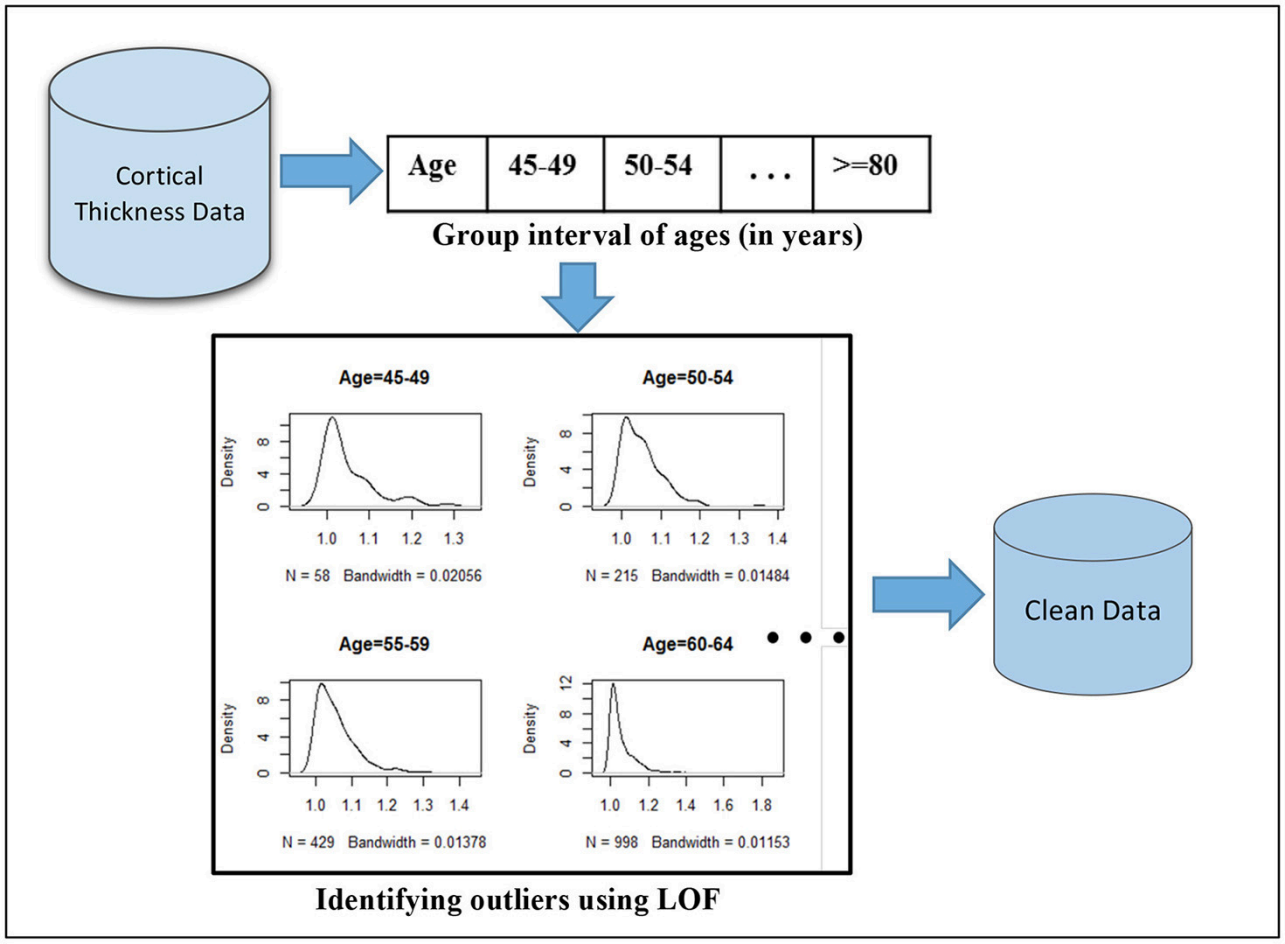
Outlier filtering approach: first, the mean cortical thickness values extracted from brain images are partitioned based on eight age interval groups and LOF algorithm is applied on each age group interval separately to compute outlier scores. The histograms illustrate the distribution of resulting outlier scores. Outliers correspond to the highest values, which are skewed to the right.

**Figure 3:**
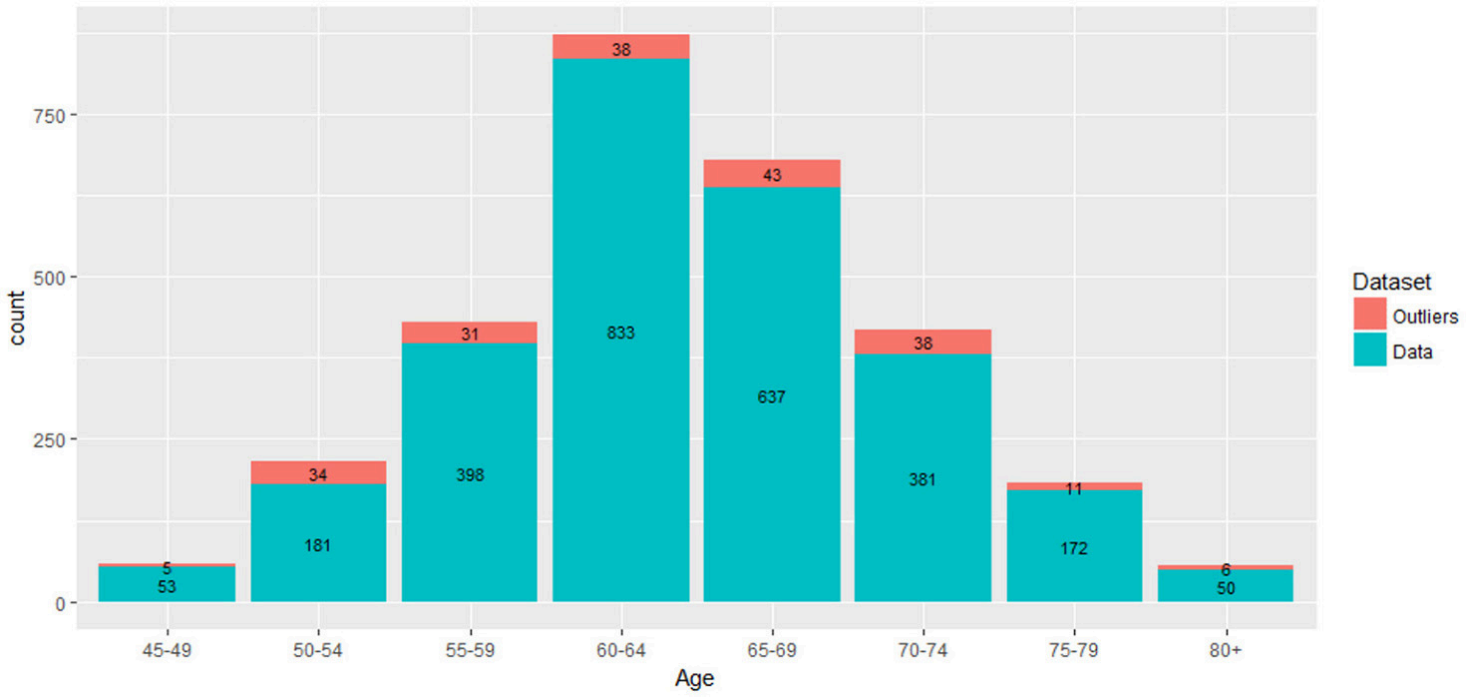
Number of samples and identified outliers per age group interval.

#### Sparse group lasso

SGL is a robust and consistent regularization model with *L*_1_ and *L*_2_ penalties for grouped variables (Chatterjee et al., 2012; Simon et al., 2013). SGL has a sparse effect both between and within groups. Sparsity is a property of learning methods that results when only a small number of coefficients of the model are non-zero. The majority of real-world problems can be sparsely represented because only a subset of the underlying features is required to best-fit a model that generalizes well to test instances. Regularization methods are frequently used to maintain the complexity of the model at a reasonable level to prevent the problem of overfitting.

Considering the regression problem of predicting brain ages of *N* individuals *Y* ∈ ℝ^*N*^ based on *X* ∈ ℝ^*N* × *P*^, we can represent the problem in multiple linear regression form as given in Equation (1).

(1) $$\begin{array}{r} Y=X\beta +\varepsilon \end{array}$$

where *Y* ∈ ℝ^*N*^ is the vector of response variable, age; *X* ∈ ℝ^*N* × *P*^ is the matrix of predicting variables; β ∈ ℝ^*P*^ is a weight vector–the unknown parameters; and ε ∈ ℝ^*N*^ is a vector of random errors. We omit the bias without loss of generality. Then the Ordinary Least Square (OLS) method estimates the parameters β* by* minimizing the cost function given in Equation (2).

(2) $$\begin{array}{r} ||Y-X\beta |{|}_{2}^{2} \end{array}$$

However, OLS has a limitation with respect to high collinearity and on high-dimensional data, including when the number of observations is less than the number of predictors. Lasso (Least Absolute Shrinkage and Selection Operator) is a regularization method that promotes sparsity by extending the OLS method with an additional penalty term (Tibshirani, 1996). Lasso estimates β by minimizing Equation (3).

(3) $$\begin{array}{r} \frac{1}{2}||Y-X\beta |{|}_{2}^{2\;}+\lambda ||\beta |{|}_{1} \end{array}$$

Further, (Yuan and Lin, 2007) developed a Group Lasso method for grouped variables. It minimizes the objective function given in Equation (4) to estimate β.

(4) $$\begin{array}{r} {\underset{\beta }{\min}\frac{1}{2}||Y-\overset{m}{\underset{l=1}{\sum }}X^{\left(l\right)}\beta^{\left(l\right)}\;||}_{2}^{2}+\lambda \overset{m}{\underset{l=1}{\sum }}\sqrt{p_{l}}||\beta^{\left(l\right)}|{|}_{2} \end{array}$$

where m is number of groups, *X*^(*l*)^ and β^(*l*)^ are predictors and coefficients in-group *l, l* = 1, 2, …, *m*, respectively and *p*_*l*_ is the length of β^(*l*)^. The Group Lasso produces a sparse set of groups that are related to the response variable. That is, if a group is selected in the model then all coefficients in the group are non-zero. SGL combines Group Lasso and Lasso methods to select the most important variables between and within groups. Considering *m* total group of predictors, the general SGL model representation is given in Equation (5).

(5) $$\begin{array}{r} \underset{\beta }{\min}\frac{1}{2n}||Y-\overset{m}{\underset{l=1}{\sum }}X^{\left(l\right)}\beta^{\left(l\right)}|{|}_{2}^{2}\\ +\left(1-\alpha \right)\lambda \overset{m}{\underset{l=1}{\sum }}\sqrt{p_{l}}||\beta^{\left(l\right)}|{|}_{2}+\;\alpha \lambda ||\beta |{|}_{1} \end{array}$$

where *X*^(*l*)^ is the predictors in-group *l*; β^(*l*)^ is the coefficient vector in-group *l, l* = 1, …, *p*_*l*_; *p*_*l*_ = the total number of features in-group *l, l* = 1, …, *m*. α* and λ* are hyper-parameters of the model. The value of α is between zero and one and controls the weight assigned to the *L*_1_and *L*_2_ penalties; That is, α = 0 produces Group Lasso model; α = 1 produces the Lasso model; when 0 < α < 1, we obtain a balance between the two schemes. In our case we used α = 0.25. An optimal estimation of the tuning parameter λ is important for prediction accuracy. We used 10-fold cross validation on λ sequences of length 50 in such a way that the optimal value of λ is the point at which an increase of λ does not provide substantial decrease of cost function.

The resulting coefficients of SGL are sparse, that is, only a small number of the coefficients are non-zero; hence, the most important features with non-zero coefficients can be automatically selected. Therefore, it supports simultaneous feature selection and regression coefficient estimation in a single framework. In the proposed approach, we used SGL using the cerebral lobe classification of the cortex structure as primary groups. Cortical regions outside of the major cerebral lobes are grouped as “Others.” Gender and education features were considered as a singleton group, i.e., their group size was one. Thus, the value of *m* in the SGL model was nine. Table 2 summarizes the details of the groupings used in our analysis. We analyzed our model using the R package SGL library (https://CRAN.R-project.org/package = SGL).

**Table 2:** Grouping list of predicting variables.

**l**	**Frontal**	**Temporal**	**Limbic**	**Parietal**	**Occipital**	**Insula**	**Others**	**Gender**	**Education**
	**1**	**2**	**3**	**4**	**5**	**6**	**7**	**8**	**9**
*p*_*l*_	38	30	16	20	20	16	8	1	1

#### Gaussian process regression

A Gaussian process (GP) is a collection of random variables, where any subset of the variables follows a multivariate Gaussian distribution (Rasmussen and Williams, 2006). GP can be used to describe probabilities over arbitrary functions, which allows us to apply it in a regression setting called Gaussian Process Regression (GPR). GPR is a non-parametric regression model based on the Bayesian approach (Rasmussen and Williams, 2006). Multivariate GP can show local patterns of covariance between individual points. Moreover, the combination of multiple Gaussians in GP can model non-linear relationships and it is more flexible than parametric models. GPR previously demonstrated high accuracy in predicting age from T1-MRI data of voxel based morphometry (Cole et al., 2015).

Considering the training dataset of input-target pairs ${\left\{x_{i},y_{i}\right\}}_{i=1}^{m}$, the GPR assumes the output *y*_*i*_ as a function *f* on input *x*_*i*_ as given in Equation (6):

(6) $$\begin{array}{r} y_{i}=f\left(x_{i}\right)+\varepsilon_{i} \end{array}$$

where $\varepsilon_{i}∽\left(0,\;\sigma^{2}\right)$. Let *Y* ∈ ℝ^*m*^ be the vector of the response variables *y*_*i*_, and *X* ∈ ℝ^*m* × *P*^ be a matrix of features. The GP for the distribution of function values we are trying to estimate are based on the mean, *m*(*X*) and a covariance function *K*(*X, X*′), as given in Equation (7).

(7) $$\begin{array}{r} f\left(x\right)∽GP\left(m\left(X\right),\;K\left(X,\;X^{\prime }\right)\right) \end{array}$$

The covariance function*K*(*X, X*′), which is called a kernel function, describes the relationship between the function values at all input points *X* and *X*′. The prior mean *m*(*X*) is usually set to zero without loss of generality, i.e., the set of function variables have a zero mean Gaussian distribution as indicated in Equation (8).

(8) $$\begin{array}{r} f\left(X\right)\sim GP\left(0,K\left(X,X^{\prime }\right)\right) \end{array}$$

For some valid covariance function *K*(*X, X*′), considering the test dataset ${\left\{x_{i}^{*},y_{i}^{*}\right\}}_{i=1}^{n^{*}}$ and the corresponding response variable vector $Y_{{\;}^{*}}={\left(y_{1}^{*},y_{2}^{*},\;\ldots \;,\;y_{n^{*}}^{*}\right)}^{T}\in {\mathbb{R}}^{n^{*}},\;$ and n^*^ × *P* matrix of features, ${\;X}_{*}={\left(x_{1}^{*},x_{2}^{*},\;\ldots \;,\;x_{n^{*}}^{*}\right)}^{T}\in {\mathbb{R}}^{n^{*}\times P}$, the prediction of the response variable Y_*_ using predicting variables X_*_ can be obtained by using the conditional Gaussian distribution given in Equation (9).

(9) $$\begin{array}{r} P\left(Y_{{\;}^{*}}|Y,\;X,X_{{\;}^{*}}\right)=P\left(f_{{\;}^{*}}|f,\;X,X_{{\;}^{*}}\right)\sim N\left(\mu^{{\;}^{*}},\Sigma^{{\;}^{*}}\right) \end{array}$$

where,

$$\begin{array}{l} \mu^{*}=K\left(X_{*},\;X\right){\left(K\left(X,X\right)+\sigma^{2}I\right)}^{-1}Y\\ \Sigma^{*}=K\left(X_{*},X_{*}\right)+\;\sigma^{2}I\;-K\left(X_{*},X\right)(K\left(X,X\right)\\ \;+\sigma^{2}I{)}^{-1}K\left(X,X_{*}\right) \end{array}$$

In addition, a special case of GPR called Relevance Vector Regression (RVR) is used for comparison as it has been widely used previously in predicting brain age from T1-MRI data (Ashburner, 2007; Franke et al., 2010; Kondo et al., 2015; Wang et al., 2015; Madan and Kensinger, 2018). RVR is a Bayesian sparse learning model used for regression and classification (Tipping, 2001). RVR determines the relationship between the target output and the covariates by enforcing sparsity. Given the training dataset of input-target pairs${\;\left\{x_{i},y_{i}\right\}}_{i=1\;}^{n},$ the prediction of the unseen data *x* can be defined as a linear combination of a kernel function as given in Equation (10).

(10) $$\begin{array}{r} f\left(x\right)=\beta_{0}+\;\overset{P}{\underset{i=1}{\sum }}\beta_{i}\Phi_{i}\left(x\right) \end{array}$$

where β = (β_0_, β_0_, …, β_*P*_) is vector of weights and Φ_*i*_(*x*) = *K*(*x, x*_*i*_) is the kernel function defining the basis function of each example of the training set. Because in the $y_{i}=f\left(x_{i};\beta \right)+\;\varepsilon_{i,\;}\;\varepsilon_{i}\;\sim N\left(0,\sigma^{2}\right)$, thus, $P\left(y_{i}|x_{i}\right)=N\left(y_{i}|y\left(x_{i}\right),\;\sigma^{2}\right)$. Accordingly, the likelihood estimate based on the Gaussian distribution is given in Equation (11).

(11) $$P\left(Y|\beta ,\sigma^{2}\right)={\left(2\pi \sigma^{2}\right)}^{\frac{-n}{2}}exp\left\{-\frac{1}{2\sigma^{2}}{\left\|y-\Phi \beta \right\|}^{2}\right\}$$

where $Y={\left(y_{1,\;}y_{2,\;\cdots ,\;}y_{N}\right)}^{T},$ and Φ is the *N* × (*N* + 1) design matrix with $\Phi ={\left[\Phi \left(x_{1}\right),\Phi \left(x_{2}\right),\ldots \;,\;\Phi \left(x_{N}\right)\right]}^{T}$ and $\Phi \left(x_{i}\right)={\left[1,K\left(x_{i},x_{1}\right),\;K\left(x_{i},x_{1}\right),\;\ldots \;,\;K\left(x_{i},x_{N}\right)\right]}^{T}$

We analyzed our model of GPR and RVR by using R package “kernlab” library (http://www.jstatsoft.org/v11/i09/). The GPR is fitted using Radial Basis Function (RBF) on the most important features selected from the *P* covariates by SGL described in section Sparse Group Lasso.

#### Deep neural network

Deep Learning was used for comparison, as it has previously shown high accuracy in predicting brain age on MRI raw data (Cole et al., 2017). An artificial neural network (ANN) is a machine learning algorithm inspired by a structure of the brain (Goodfellow et al., 2016). Architecturally, the neurons of ANN are interconnected and arranged in input, hidden, and output layers. ANN is typically classified as single layer or multilayer based on the number of hidden layers. Neural nets having only one hidden layer are called shallow neural networks. Multilayer neural networks, i.e., having two or more hidden layers, are called deep neural networks (DNNs) or deep learning. DNN is very helpful in automatic feature learning from complex non-linear data representations. Autoencoder is a special type of DNN architecture that can be used for feature extraction. It is an unsupervised learning method that attempts to reconstruct its input (Goodfellow et al., 2016). Technically, autoencoders are feedforward neural networks with one hidden layer where the input is the same as the output. In other words, the input is compressed to lower dimensional code representation called a hidden layer and the output is reconstructed from this representation. The objective of the autoencoder is to learn an efficient and compact hidden representation of the input to successfully reconstruct it. Both the encoder and the decoder functions employ a form of non-linearity in order to learn rich representations. A stacked auto-encoder (SAE) is a DNN consisting of multiple layers of sparse autoencoders (Bengio et al., 2007). The outputs of each layer are wired as the inputs of the next layer. SAEs are trained in an unsupervised, greedy, and layer-wise fashion. That is, once the first layer is pre-trained as in an autoencoder by freezing all the other layers, its output is wired as input to the next hidden layer. This layer-wise training is continued to the last layer.

In brain age estimation, we used *H*_2_O package in R (https://github.com/h2oai/h2o-3) for the analysis of DNN model. The rectified linear unit (ReLU) activation function and dropout regularization methods are used to train the DNN model. The number of hidden layers is four with 94, 48, 48 and 94 neurons in each hidden layer, respectively. In addition, we used stacked autoencoder to extract features from the *P* = 150 covariates irrespective of the cortical lobe grouping structure. In SAE, it takes the designated covariate vector *X* ∈ ℝ^*P*^ and maps to the deep hidden vector representation *h* ∈ ℝ^*P*′^, *P*′ < *P*. The extracted features (vector *h* ∈ ℝ^*P*′^) are used as input to fit the regression models for comparison with the SGL approach. For SAE, the number of hidden layers is two with 94 and 61 neurons in each layer, respectively. One of the challenges of deep learning is that the learning requires a very large dataset in order to obtain adequate prediction accuracy.

#### Cross validation

The dataset (*N* = 2,705) was stratified based on age and randomly divided into training set and test set. About 70% of the dataset was used for the training set (*N*_1_ = 1,895) and the remaining 30% used for the test set (*N*_2_ = 810). The 10-fold cross validation was done on the training set. We repeated this train-test split 10 times to obtain reliable generalizations. For each of the ten repetitions, the *N* observations were randomly resampled and divided into training set and test set. Then, the training set was randomly split into 10-folds for cross validation. When the first fold was used as the validation set, the model is fit on the remaining 9-folds. The mean square error (*MSE*_1_) was then computed for the held-out fold. For each fold, the MSE was computed as given in Equation (12).

(12) $$\begin{array}{r} MSE_{k}=\;\frac{1}{n}\sum_{i=1}^{n}{\left({ŷ}_{i}-y_{i}\right)}^{2},\;k=1,2,\ldots \;,\;10. \end{array}$$

where *y*_*i*_ is the actual age, ŷ_*i*_ is the predicted age, and *n* is number of subjects in the validation set of a given fold. The 10-fold cross validation estimate (CVE) was computed by averaging Equation (12) as shown in Equation (13).

(13) $$\begin{array}{r} CVE=\frac{1}{10}\sum_{k=1}^{10}MSE_{k}\; \end{array}$$

All performance assessment results reported for the brain age prediction model were conducted on the test set. Thus, for each resampled test dataset, we computed accuracy of age estimation in terms of RMSE and MAE. The RMSE and MAE can be computed as given in Equations (14,15).

(14) $$\begin{array}{r} RMSE_{j}=\sqrt{\frac{1}{N_{2}}\sum_{i=1}^{N_{2}}{\left({ŷ}_{i}-y_{i}\right)}^{2}} \end{array}$$

(15) $$\begin{array}{r} MAE_{j}=\frac{1}{N_{2}}\sum_{i=1}^{N_{2}}|{ŷ}_{i}-y_{i}| \end{array}$$

where *N*_2_ is the number of samples in the test set of each repeat. Finally, the generalization accuracy of the model is given as the average of Equations (14,15) as given in Equations (16,17).

(16) $$\begin{array}{r} RMSE=\frac{1}{10}\sum_{j=1}^{10}RMSE_{j}\; \end{array}$$

(17) $$\begin{array}{r} MAE=\frac{1}{10}\sum_{j=1}^{10}MAE_{j}\; \end{array}$$

## Results and discussion

### Effect of outliers

The dataset in this study included only cognitively normal subjects. The objective of outlier checking was to investigate outliers that could violate this condition because our samples were from older adults (age 45–91 years), which are in a risk zone of cognitive disorder. Outliers affect the prediction accuracy of the model when non-robust statistical methods are used. As described in section Filtering Outliers From Cortical Thickness Data, we designed an algorithm that best suits the mean cortical thickness data representation by adopting an LOF method. The proposed outlier filtering approach showed a slight performance improvement in all tested models, as displayed in Table 3. The performance results of RMSE and MAE are in years and computed on the test dataset.

**Table 3:** Performance results before and after filtering outliers.

**#**	**Model**	**All data**		**After removing outliers**	
		**RMSE**	**MAE**	**RMSE**	**MAE**
1	OLS	5.409	4.240	5.264	4.112
2	SGL	5.347	4.162	5.265	4.071
3	GPR	5.274	4.151	5.139	4.033
4	RVR	5.368	4.213	5.179	4.063
5	DNN	5.378	4.181	5.160	4.022

We compared the generalization results of different regression models on the dataset after removing outliers (resulting in *N* = 2,705 samples). The models were trained using 10-fold cross-validation repeated 10 times for reliability of the performance results. The best accuracy result was obtained by GPR model (RMSE = 5.18 years and MAE = 4.08 years) as shown in Table 4. All other models also showed comparable results.

**Table 4:** Performance comparison of different regression models.

**#**	**Model**	**RMSE**	**MAE**
1	OLS	5.296	4.206
2	SGL	5.281	4.145
3	GPR	5.184	4.078
4	RVR	5.241	4.127
5	DNN	5.268	4.137

### Performance of hybrid methods

As described in section Sparse Group Lasso, we used the SGL model to select the most important features based on macro-anatomical cortex structure grouping of the brain. The major benefit of SGL is its robustness to select features on-group and within-group levels. The grouping of brain structures according to cerebral lobes best complies with the SGL benefits. In our dataset we had *N* = 2,705 observations and *P* = 150 covariates (ROIs = 148 and demographic variables–gender and education). Among the *P* = 150 covariates, the SGL method selected 94 features (ROIs = 93 and Education). Gender was not selected as an important predictor by SGL. Accordingly, we applied RVR and GPR on the selected features to estimate the brain age. The combination of SGL and GPR showed an improved performance result (RMSE = 5.16 years and MAE = 4.05 years) as indicated in Table 5. The paired *t*-test between MAEs from SGL + GPR and SGL + RVR produced a *p*-value of 0.004, which shows the statistical significance of the performance difference. The results from GPR and SGL + GPR also showed significant difference with *p*-value of 0.003.

**Table 5:** Performance results of hybrid approaches; SD, standard deviation.

**#**	**Hybrid methods**	**RMSE**		**MAE**	
		**Mean**	**SD**	**Mean**	**SD**
1	SGL + GPR	5.157	0.119	4.053	0.099
2	SGL + RVR	5.191	0.108	4.094	0.092
3	SAE + GPR	5.185	0.159	4.063	0.133
4	SAE + RVR	5.266	0.137	4.135	0.112

In addition, we tested stacked autoencoder (SAE) for feature extraction based on the ungrouped covariates. The SAE was used to extract the features, and we applied RVR and GPR to estimate the brain age. The results were comparable with that of SGL combined with GPR. However, if the actual selected features need to be known, it is impossible to identify them in the case of SAE as the learning of features is weighted and combined. This is because SAE learns a combination of features, whereas we were attempting to select a subset of features using SGL. The results of the hybrid methods are presented in Table 5.

The overall results of the SGL and GPR hybrid model showed marginal improvement over GPR. In addition, with SGL, the most important cortex regions are identified. Many studies showed that late life education reduces the rate of cortical thinning (Belathur Suresh et al., 2018; Thow et al., 2018). Thus, education can be considered as one of the features for brain age prediction.

The value of *R*^2^ we obtained across the different methods is around 0.5 which is relatively lower than previous works. We conjecture this might be partly due to the age range of our study population. In this work, we studied only older adults (age 45–91 years with mean age around 64) than in other studies. Moreover, owing to the more complex non-linear relationship between normal aging and cortex anatomical structure of older adults, the amount of variance in the dependent variable explained by the independent variable(s) could be smaller. Therefore, *R*^2^ may not always be an absolutely better measure that could compare the results under different age range and sample size. We would like to emphasize that our method has the additional advantage over previous methods in that it identifies the most contributing features and cortex regions having cortical thinning pattern due to normal aging.

**Figure 4:**
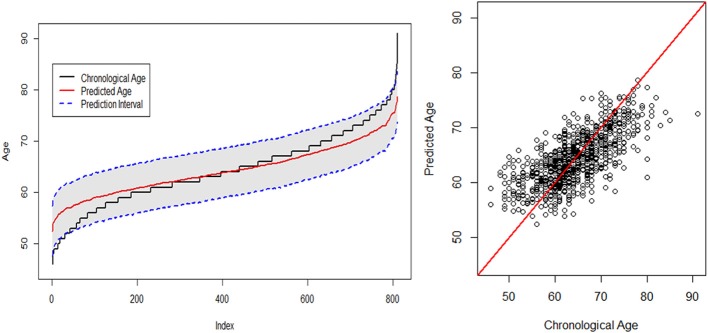
Chronological age vs. predicted age using the SGL + GPR model.

The fitted plots for the SGL + GPR model are presented in Figure 4. The plot on the left displays the fitted lines of both chronological age and predicted age indexed from the least value of actual age to the greatest value. For example, the index for age 45 is “1,” and the index for age 91 is *m* = 810 where *m* is the number of samples in the test set. Accordingly, the prediction interval of the test sample is shown as the gray area in Figure 4 (left). The scatter plot for the chronological age vs. the predicted age is shown on the right in Figure 4. We find that the estimation result reveals an age-related bias such that the predicted age is higher for younger subjects and lower for older subjects. This appears to be due to the sample size imbalance across age groups. The prediction of age tends to be more accurate where more samples exist for each age, particularly in the 50–79 range, and the estimations for those groups in two extreme ranges with fewer samples tend to be substantially influenced by the estimates for the majority of samples in the middle. See Table 1 for the detailed distribution of the ages in the target samples. A similar observation and discussion has been presented in Pardoe and Kuzniecky (2018).

### Consistency of SGL

We used a resampling method in order to verify the consistency of the SGL feature selection. We trained the SGL model with ten different random sample combinations of our dataset to validate its consistency. The experimental results showed that the majority of features selected were also repeatedly selected in the 10 random trials. Among the 94 features selected by SGL, 61 of them were repeatedly selected in different resampling trials. Education was selected in all ten trials. Gender was not selected in any of the ten trials. Among the 148 ROIs, most of the repeatedly selected cortex regions were from the frontal lobe, temporal lobe, and parietal lobe. The results of age estimation on these repeatedly-selected features showed comparable results with the 94 features. This shows the credibility of the selected regions, indicating the regions having cortical thinning patterns associated with normal aging. The results of age estimation on the repeatedly selected 61 features are shown in Table 6.

**Table 6:** Age estimation on repeatedly selected 61 features; the number of repetition is ten.

**#**	**Hybrid methods**	**RMSE**		**MAE**	
		**Mean**	**SD**	**Mean**	**SD**
1	SGL + GPR	5.187	0.107	4.074	0.087
2	SGL + RVR	5.191	0.091	4.083	0.076
3	SAE + GPR	5.203	0.104	4.081	0.082
4	SAE + RVR	5.282	0.088	4.157	0.074

### Analysis of the proposed model

**Figure 5:**
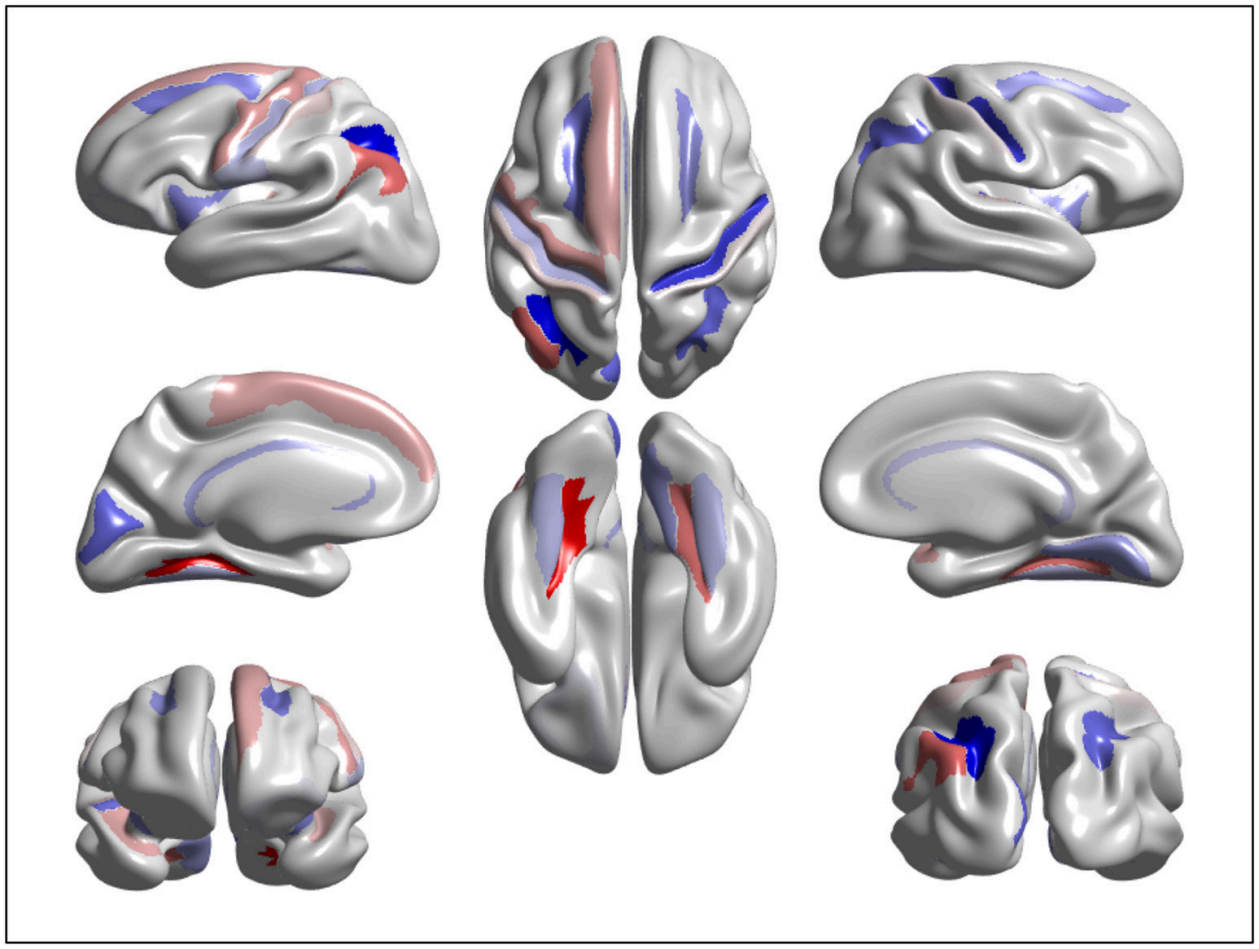
Visualization of the features significantly contributing to age estimation: the warm and cold color represent important features in predicting brain age using cortical thinning patterns.

We designed a brain age prediction model with cortical thickness data extracted from 148 ROIs and confounding demographic variables of gender and education. The proposed method used SGL to select the most important features contributing to brain age prediction. SGL selected 94 features i.e., 93 ROIs and Education. The selected cortex regions are shown in Figure 5 with different colors for cortical lobe groupings. The majority of the selected regions are from cortex regions having cortical thinning pattern associated to normal aging (Allen et al., 2005; Fjell et al., 2009; Lee et al., 2016a,b). Our target study age range is from 45 to 91. In this range, brain cortical thickness declines due to normal aging. Education is one of the most important factors that reduce the rate of cortical thinning (Thow et al., 2018). Thus, Education has a contribution to the prediction of brain age as a confounding factor. We used these selected features to predict brain age using a GPR model and obtained good performance accuracy of MAE = 4.05 years. The combination of SGL with GPR has two benefits. First, it identifies the most contributing cortex regions having cortical thinning pattern due to normal aging. Secondly, it offers a comparable or superior generalization result to GPR alone or other models.

The importance of surface-based morphology in comparison to voxel-based morphology was reviewed by Mechelli et al. (2005), Clarkson et al. (2011), and Madan and Kensinger (2018). The surface-based approach allows examining distinct measures of cortical structure. In contrast, the volume-based approach that estimates gray matter volume is influenced by a combination of structural features. In surface-based morphology, the predictive value of each specific ROI's cortical thickness can be assessed.

For an insightful analysis of our approach in relation to related studies of age prediction from brain anatomical features, the comparison of our model with the state-of-the-art studies is presented in Table 7. The prediction accuracy of our approach is comparable with the recent state-of-the-art studies. The major contribution to the improvement of generalization accuracy is the SGL regularization technique used to select important features that best fit the age prediction model. It is worth mentioning that each previous study for comparison had slightly different experimental setting because they either used a small sample size or studied relatively young subjects (see the mean age of the previous studies in Table 7). In this work, we studied only older adults (age 45–91 years). In this older age lifespan, the risk of dementia is higher. Cognitive functioning declines due to normal aging. Moreover, owing to the more complex non-linear relationship between normal aging and cortex anatomical structure of older adults, the amount of variance in the dependent variable explained by the independent variable(s) could be different.

**Table 7:** Our model vs. related studies: N, number of samples; ^*^ given in median absolute error (MdAE).

**#**	**Author**	**Age range**	**Mean age**	***N***	**Model**	**RMSE**	**MAE**
1	Ashburner, 2007	17–79	31.80	471	RVR	6.50	–
2	Franke et al., 2010	19–86	48.08	547	RVR	5.90	4.61
3	Wang et al., 2015	20–82	47.04	360	RVR	5.57	4.57
4	Kondo et al., 2015	20–75	45.60	1,146	RVR	5.65	4.50
5	Cole et al., 2015	18–90	–	1,749	GPR	–	4.66
6	Cole and Franke, 2017	18–90	36.95	2,001	CNN	5.31	4.16
7	Madan and Kensinger, 2018	18–97	–	1,056	RVR	–	6–7^*^
8	Our model	45–91	64.20	2,705	SGL + GPR	5.16	4.05

The cortical thickness measurements are known to be sensitive to the selection of scanner vendor, imaging protocols, or sites. Our dataset was collected from one center (SMC) using a single scanner with the same protocol which is one of the limitations to consider. In addition, our study aims to model the biological age from healthy subject samples, and therefore the analysis of cortical thinning due to pathology is out of the scope. The other related point is atrophy in the subcortical structures might be important in normal aging. Our study focuses on the cortical change due to normal aging; the atrophy in the subcortical structures can be considered in the future study.

### Analysis of brain features significantly contributing to age estimation

We extracted and visualized the brain features that significantly contribute to the age estimation. Figure 5 shows a cortical thinning pattern specifically associated with normal aging, extracted by the SGL model. As shown in the figure, our findings were consistent with previous studies. A recent study from our group suggested that there were brain regions vulnerable to brain aging. Specifically, compared to those in their twenties and thirties, participants in their forties showed thinning primarily in the medial and lateral frontal and inferior parietal regions, and cortical thinning occurred across most of the cortices with increasing age (Lee et al., 2016a,b).

We find that age-related cortical thinning occurs on areas responsible for executive processing tasks, spatial cognition, vocabulary learning, and episodic memory retrieval, which are also known to be associated with age-related cognitive decline(Pochon et al., 2001; Monsell, 2003; Cavanna and Trimble, 2006; Singh-Curry and Husain, 2009; Caspers et al., 2012; Barbey et al., 2013). Furthermore, our findings could support the “last in, first out” hypothesis(McGinnis et al., 2011). That is, late-maturing regions of the brain, such as the heteromodal association cortices, are preferentially vulnerable to age-related loss of structural integrity.

## Conclusions

We presented a brain age estimation model using cortical thickness data extracted from T1 MRI. We designed a feature selection approach that identifies cortical regions associated with cortical thinning and better generalizes brain age prediction. The best prediction accuracy was obtained with the SGL + GPR hybrid model. The best performance result was MAE = 4.05 years, which is comparable with results obtained by several recent state-of-the-art studies. The deep learning automatic feature-learning capability of the stacked auto-encoder also showed comparable result when combined with GPR. In general, the analysis of this research indicates the desirability of feature selection strategies to design predictive models of brain age from surface-based features that are capable of generalizing.

## Author contributions

J-KS, SS, and K-AS designed the main idea and directed the overall analysis. HA, J-HS, and BK developed the algorithm and carried out the data processing and experiments. DN and SS helped the data collection and result interpretation. HA, J-KS, SS, and K-AS wrote the manuscript with input from all authors.

### Conflict of interest statement

The authors declare that the research was conducted in the absence of any commercial or financial relationships that could be construed as a potential conflict of interest.
